# Hyperglycemia secondary to phosphatidylinositol-3 kinase (PI3K) inhibition

**DOI:** 10.1530/EDM-24-0040

**Published:** 2024-10-21

**Authors:** Arunan Sriravindrarajah, Joshua Hurwitz, Elgene Lim, Jerry R Greenfield

**Affiliations:** 1 St Vincent’s Hospital, Sydney, Australia; 2 School of Clinical Medicine, UNSW Medicine & Health, St Vincent's Healthcare Clinical Campus, Faculty of Medicine and Health, UNSW, Sydney, Australia; 3 Garvan Institute of Medical Research, Sydney, Australia; 4 The Kinghorn Cancer Center, Sydney, Australia

**Keywords:** PI3K inhibitor, hyperglycemia, Diabetes, Please select country of treatment for each patient reported, AUSTRALIA

## Abstract

**Summary:**

Phosphatidylinositol-3 kinase (PI3K) is a critical intracellular pathway that regulates cell growth, metabolism, and survival and has been implicated in most human cancers. Targeting this pathway has been approved as a therapeutic option for breast cancer and lymphoma (e.g. alpelisib, idelalisib), and there are several clinical trials underway in additional types of cancer. However, PI3K is an important mediator of the action of insulin, and the use of PI3K inhibitors has been associated with hyperglycemia. We report the case of a 53-year-old female with metastatic breast cancer who developed acute grade 3 hyperglycemia from a novel PI3K inhibitor, inavolisib. We review the treatment options for PI3K inhibitor-associated hyperglycemia. Treatment strategies that minimize hyperinsulinemia may be preferable considering animal models have demonstrated that hyperinsulinemia may result in partial reactivation of the PI3K pathway and counter the anti-cancer effectiveness of PI3K inhibitors.

**Learning points:**

- Phosphatidylinositol-3 kinase (PI3K) is an intracellular pathway that regulates a range of physiological functions, including cell growth, metabolism, survival, and angiogenesis.
- Hyperactivation of the PI3K pathway is associated with almost all human cancers, and thus PI3K inhibition has been proposed as a treatment option for selected cancers.
- The action of insulin after binding to the insulin receptor on the cell surface (e.g. glucose uptake in skeletal muscle, inhibition of glycogenolysis and gluconeogenesis) is mediated by the intracellular PI3K pathway, and thus PI3K inhibition may lead to hyperinsulinemic hyperglycemia.
- All patients treated with PI3K inhibitors should receive pre-treatment screening for hyperglycemia, lifestyle advice, and a glucometer to measure fasting BGL and 2-h post-dinner BGL levels twice per week for at least the first 30 days of treatment.
- Insulin or insulin secretagogues (e.g. sulfonylurea) may inhibit the anti-tumor activity of PI3K inhibitors, and thus treatment of PI3K inhibitor-associated hyperglycemia should prefer alternative approaches such as a low carbohydrate diet, metformin, SGLT2i, or dose reduction of the PI3K inhibitor.

## Background

Phosphatidylinositol-3 kinase (PI3K) is a critical intracellular pathway that regulates cell growth, angiogenesis, metabolism, and survival (1). Activation of PI3K and its downstream signaling kinases protein kinase B (AKT) and mammalian target of rapamycin (mTOR) has been associated with oncogenesis and the development of resistance to other anti-cancer therapies. For example, ~40% of patients with advanced hormone receptor-positive, human epidermal growth factor receptor 2-negative (HER2–) breast cancer demonstrate activating mutations in the PI3K catalytic subunit alpha (PIK3CA) gene, which encodes the p110α-isoform of PI3K (1, 2). The PI3K/AKT/mTOR pathway has been found to be dysregulated in most human cancers, especially breast cancer, and targeting this pathway has been proposed as a potential therapeutic option (1). However, PI3K is an important mediator of the action of insulin, and the use of PI3K inhibitors has been associated with hyperglycemia as an ‘on-target’ side effect (2). The phase 3 SOLAR-1 trial of alpelisib, a PIK3CA inhibitor, demonstrated hyperglycemia as the most common adverse event occurring in 63.7% of patients, with grade 3 or 4 hyperglycemia (blood glucose level (BGL) >250 mg/dL or 13.9 mmol/L) observed in 36.6% of patients (3). We report the case of a 63-year-old woman with metastatic breast cancer, without pre-existing diabetes mellitus, who developed marked hyperglycemia after commencement of a novel PIK3CA inhibitor and review treatment options for PI3K inhibitor-associated hyperglycemia.

## Case presentation

A 63-year-old Pacific Islander female, with no personal or family history of diabetes mellitus or recent glucocorticoid usage, was referred to the Diabetes Center for management of new-onset hyperglycemia of 439.2 mg/dL (24.4 mmol/L) following 2 weeks of treatment with inavolisib, a trial drug inhibiting the PIK3CA pathway, and fulvestrant. The patient had metastatic breast cancer with progressive disease involving the liver, bone, soft tissue, and lymph nodes and prior lines of treatment with adjuvant chemotherapy, adjuvant endocrine therapy, and CDK4/6 inhibition in combination with a selective estrogen receptor degrader for metastatic disease.

## Investigation

Physical examination demonstrated a body mass index (BMI) of 29.1 kg/m^2^. Investigations performed prior to the commencement of inavolisib 9 mg daily demonstrated an HbA1C of 35 mmol/mol (5.3%) and a fasting BGL of 86.4 mg/dL (4.8 mmol/L). CT imaging did not identify any pancreatic lesions.

## Treatment

Routine BGL testing was commenced in conjunction with the commencement of inavolisib. By Day 7 post-commencement of inavolisib, glycemic control deteriorated with fasting BGL elevated at 180 mg/dL (10 mmol/L), and metformin 500 mg twice daily (maximum renally adjusted dose due to chronic kidney disease stage 3B) was commenced. She subsequently departed on a 1-week overseas holiday where she continued inavolisib and metformin but discontinued blood glucose monitoring and consumed a higher carbohydrate diet. Upon her return on Day 18 post-commencement of inavolisib, her glycemic control was found to have worsened with grade 3 hyperglycemia present due to pre-prandial BGL of 360-540 mg/dL (20–30 mmol/L). Upon discussion with Endocrinology, she commenced insulin glargine 10 units daily as an outpatient to stabilize glycemia. However, hyperglycemia continued, and the patient self-discontinued both inavolisib and insulin therapy on Day 27, following which her fasting BGL normalized to 106.2 mg/dL (5.9 mmol/L) within 72 h.

Upon recommencement of inavolisib on Day 31 post initial commencement of inavolisib, BGLs increased with fasting BGLs 126–162 mg/dL (7–9 mmol/L) and pre-dinner BGLs 234–378 mg/dL (13–21 mmol/L). She was recommended to reduce carbohydrate intake and commenced insulin glargine 10 units daily but continued to experience hyperglycemia. Inavolisib was again self-ceased on Day 33 with pre-lunch BGLs normalizing within 24 h from 266.4 mg/dL to 97.2 mg/dL (14.8 mmol/L to 5.4 mmol/L). Given the recurrent episodes of Grade 2 and 3 hyperglycemia, a 33% dose reduction of inavolisib to 6 mg daily on Day 36 was implemented per the clinical trial protocol. The patient themselves then made a further dose reduction to inavolisib 3 mg daily.

## Outcome and follow-up

Following the inavolisib dose reduction, her glycemic control normalized with BGLs consistently <144 mg/dL (8 mmol/L) on metformin alone. The patient had a progression of her liver metastases on imaging at Day 60 following the start of inavolisib, which was then ceased as per the trial protocol. She was subsequently enrolled in another clinical trial involving targeted therapy with an antibody-drug conjugate. Dexamethasone 4 mg daily was also commenced on Day 98 for abdominal pain secondary to liver capsule distension from enlarging liver metastases. Her glycemic control remained stable with BGLs 86 mg/dL to 130 mg/dL (4.8 mmol/L to 7.2 mmol/L) on glucocorticoids.

## Discussion

This case demonstrates the impact of PI3K inhibitors on glucose metabolism. The commencement of inavolisib, a small molecule designed to selectively inhibit mutant PIK3CA, immediately resulted in grade 3 hyperglycemia that persisted despite the commencement of metformin and insulin glargine. Similar to previous case reports, normalization of glucose levels occurred after cessation of the PI3K inhibitor, and subsequently via a dose reduction in the PI3K inhibitor (4). Glycemic control has been maintained since the permanent cessation of the PI3K inhibitor despite the subsequent commencement of medium doses of glucocorticoids.

The PI3K pathway is a critical mediator of the glucose-lowering actions of insulin. The binding of insulin with the insulin receptor triggers a downstream cascade that activates PI3K. Activated PI3K triggers the conversion of phosphatidylinositol 4,5-bisphosphate (PIP2) to phosphatidylinositol 3,4,5-trisphosphate (PIP3), which in turn phosphorylates and activates AKT. Activated AKT then induces the translocation of glucose transporters 1 and 4 (GLUT1 and GLUT4) to the plasma membrane to trigger glucose uptake into skeletal muscle and adipose tissue (2, 5). Activated AKT also prevents the nuclear localization of the transcription factor FOXO1, leading to the inhibition of gene transcription needed for gluconeogenesis. Furthermore, AKT promotes glycolysis via stimulating hexokinase and phosphofructokinase-2 and glycogenesis via inhibiting glycogen synthase kinase 3 (GSK3) (5). Thus, inhibition of the PI3K pathway may result in hyperglycemia due to preventing glucose uptake in skeletal muscle and adipose tissue, increasing gluconeogenesis, and increasing hepatic glycogenolysis, which results in elevated BGLs and a consequent release of insulin (2).

Hyperglycemia is the most common adverse event within the PI3K inhibitor class of medication and occurred in 63.7% of patients treated with alpelisib, a PIK3CA inhibitor, in the SOLAR-1 trial (3). All patients receiving treatment with a PI3K inhibitor should be screened with a fasting BGL and HbA1C (2). Patients with diabetes mellitus (i.e. fasting BGL ≥126 mg/dL (7.0 mmol/L) or HbA1C >48 mmol/mol (6.5%)), pre-diabetes (i.e. fasting BGL 100–125 mg/dL (5.6–6.9 mmol/L) or HbA1C 39-47 mmol/mol (5.7–6.4%) and obesity (BMI >30 kg/m^2^) should be referred to an Endocrinologist for optimization of glycemic status and ongoing care. A recent single-center retrospective cohort study of 491 patients treated with PI3K and/or AKT inhibitors identified BMI >25 kg/m^2^ and HbA1C > 5.7% as independent predictors of hyperglycemia in a multivariate regression model (6). Nonetheless, the risk of hyperglycemia is high even in patients with normal baseline glycemia (7). All patients receiving treatment with a PI3K inhibitor should receive dietary advice to consume a balanced diet, minimize simple carbohydrates and spread carbohydrate intake throughout the day. Patients should also be educated regarding the signs and symptoms of hyperglycemia and advised to seek medical attention if they develop (2). All patients should be provided with a glucometer and advised to measure fasting BGL and 2-h post-dinner BGL levels twice per week for at least the first 30 days of treatment with a PI3K inhibitor, as PI3K inhibitor-associated hyperglycemia tends to occur early in the treatment course; the median time of onset for hyperglycemia was 15 days in the SOLAR-1 trial (7). Patients with fasting BGL levels ≥126 mg/dL (7.0 mmol/L) or 2 h post prandial BGL levels ≥200 mg/dL (11.1 mmol/L) should be referred to an Endocrinologist for management of hyperglycemia.

Animal models have suggested that the compensatory hyperinsulinemia occurring with PI3K inhibitor-associated hyperglycemia may result in partial reactivation of the PI3K/AKT/mTOR pathway and counter the anti-cancer effectiveness of PI3K inhibitors (8). Conversely, treatment approaches that reduce serum insulin levels (e.g. ketogenic diet, sodium glucose co-transport 2 inhibitor (SGLT2i)) enhance the anti-tumor effects of PI3K inhibitors (8). Thus, treatment of hyperinsulinemic hyperglycemia arising from the use of PI3K inhibitors is recommended to prioritize treatment strategies that will reduce insulin levels. Lifestyle approaches, such as consuming a lower carbohydrate diet and maintaining an active lifestyle, should be implemented in all patients. Metformin is first-line pharmacological therapy, whilst second-line agents include SGLT2i or thiazolidinediones.

The specific treatment approach for PI3K inhibitor-associated hyperglycemia is challenging, with mixed evidence and risks associated with the various options. Some pre-clinical studies have suggested inferior outcomes when treated with a combination of ketogenic diets and PI3K inhibitors, such as increased tumor growth in some cancers; thus, ketogenic diets are currently not recommended (8). Whilst SGLT2i have consistently shown effectiveness in the treatment of PI3K inhibitor-associated hyperglycemia, there have also been multiple case reports of diabetic ketoacidosis in the context of PI3K inhibitor use, both with and without SGLT2i usage, and thus the use of SGLT2i in this setting must be with caution (9). Insulin and insulin secretagogues (e.g. sulfonylurea) are recommended to be limited as rescue therapy for grade 3 hyperglycemia or when hyperglycemia is not controlled on multiple oral hypoglycemic agents (2). This case also demonstrates the dose-dependent and reversible nature of PI3K inhibitor-associated hyperglycemia, and thus the effectiveness of PI3K inhibitor cessation or dose reduction to mitigate hyperglycemia. Whether dose reduction limits the anti-cancer effectiveness of the treatment needs to be considered.

A limitation of this case is distinguishing the impact of insulin commencement with self-cessation of inavolisib. However, based on the limited glucose monitoring data available, the commencement of insulin had minimal improvement in glycemic levels compared to cessation or dose reduction of inavolisib. Further research is required to understand the impact of different treatment options on PI3K inhibitor-associated hyperglycemia, as well as its effect on the progression of the underlying malignancy. For instance, the phase 2 TIFA trial compare a ketogenic diet (<50g carbohydrate daily), a low carbohydrate diet (<100g carbohydrate daily), and SGLT2i for the treatment of hyperglycemia in patients with metastatic PIK3CA-mutant breast cancer on alpelisib (10).

This case demonstrates the potent hyperglycemic effect of PI3K inhibition in a patient without a history of diabetes mellitus. Management of the resulting hyperinsulinemic hyperglycemia is challenging. Monitoring BGLs from commencement, early referral to endocrinologists, commencement of hypoglycemic agents when BGLs are elevated, and co-management between oncologists and endocrinologists of patients on this class of drugs is critical for the optimal care and outcomes of these patients.

## Declaration of interest

The authors declare that there is no conflict of interest that could be perceived as prejudicing the impartiality of the study reported.

## Funding statement

This research did not receive any specific grant from any funding agency in the public, commercial or not-for-profit sector.

## Patient consent

Written informed consent for publication of their clinical details was obtained from the patient.

## Author contributions statement

All authors made individual contributions to authorship. AS, JH, EL and JRG were involved in the diagnosis and management of the patient and manuscript submission. All authors reviewed and approved the final draft.
